# Supplementary material for: Aliphatic Quaternary Ammonium Functionalized Nanogels for Gene Delivery
Source: Pharmaceutics. 2021 Nov 19;13(11):1964. doi: 10.3390/pharmaceutics13111964 (PMC8618000; doi:10.3390/pharmaceutics13111964)
Supplement: Supplementary file 1 [file pharmaceutics-13-01964-s001.zip › pharmaceutics-1451695-SI.pdf]

pharmaceutics

Supplementary Materials: Aliphatic Quaternary Ammonium Functionalized Nanogels for Gene Delivery

Huaiying Zhang, Damla Keskin, Willy H. de Haan-Visser, Guangyue Zu, Patrick van Rijn and Inge S. Zuhorn

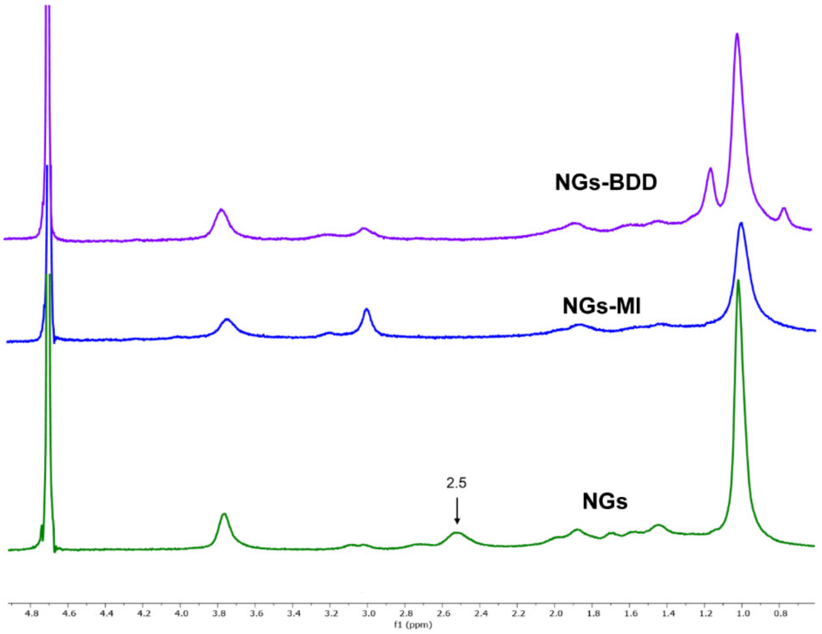

Figure S1. ^{1}H NMR spectra of NGs, NGs-MI and NGs-BDD.

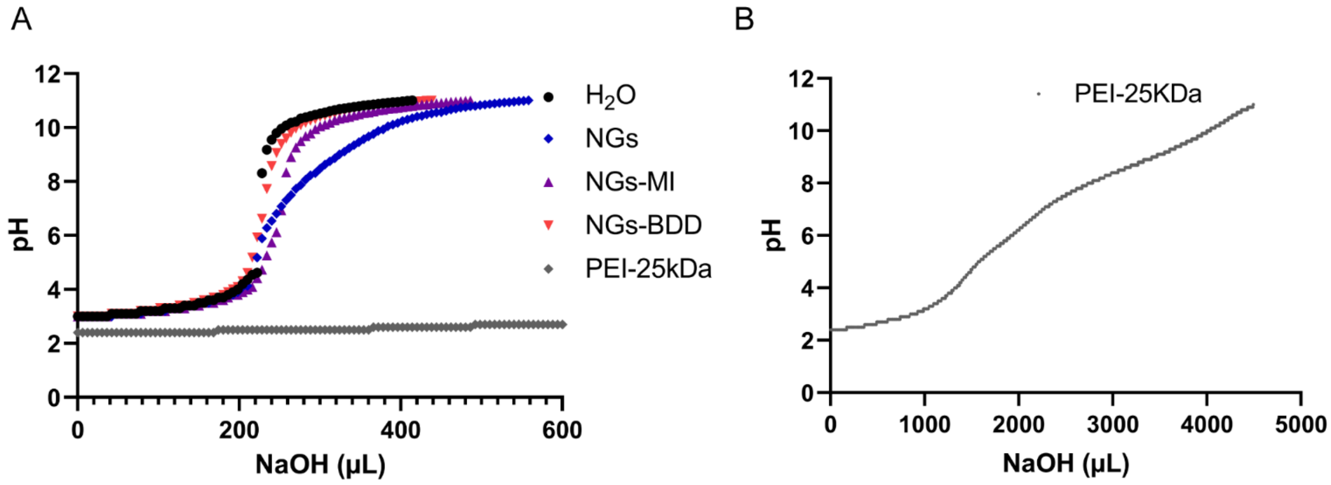

Figure S2. (A) Acid-base titration profiles of nanogels, (B) Acid-base titration profile of PEI-25kDa.

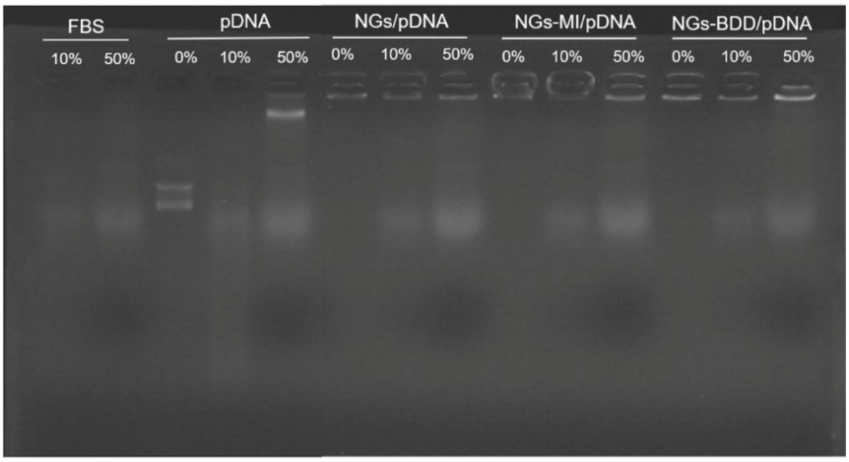

Figure S3. DNA protection against serum nucleases in nanogel/pDNA complexes. Agarose gel electrophoresis of NGs/pDNA, NGs-MI/pDNA and NGs-BDD/pDNA treated with 10% or 50% serum for 10 h.

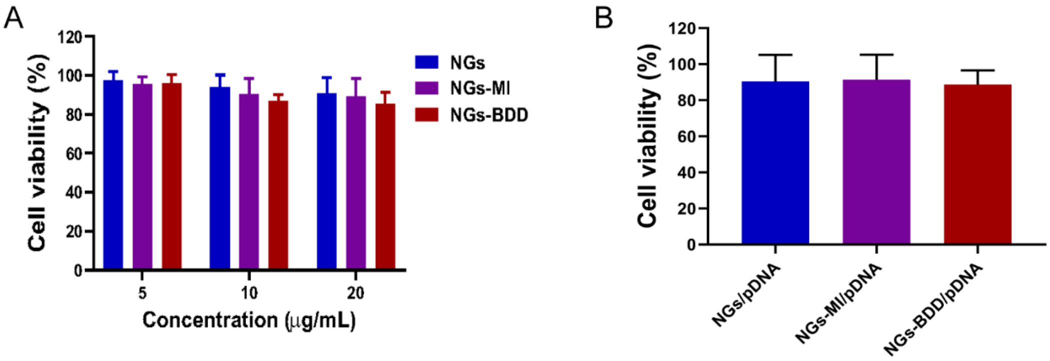

Figure S4. Cytotoxicity of nanogels and nanogel/pDNA complexes in HEK293T cells. (A) Relative viability of HEK293 cells after incubation with various concentrations of nanogels for 48 h and (B) Relative viability of HEK293 cells treated with nanogel/pDNA complexes (weight ratio 10) in opti-MEM medium containing 1 μg pDNA for 8 h, followed by culture with cell culture medium for another 40 h (n=3).

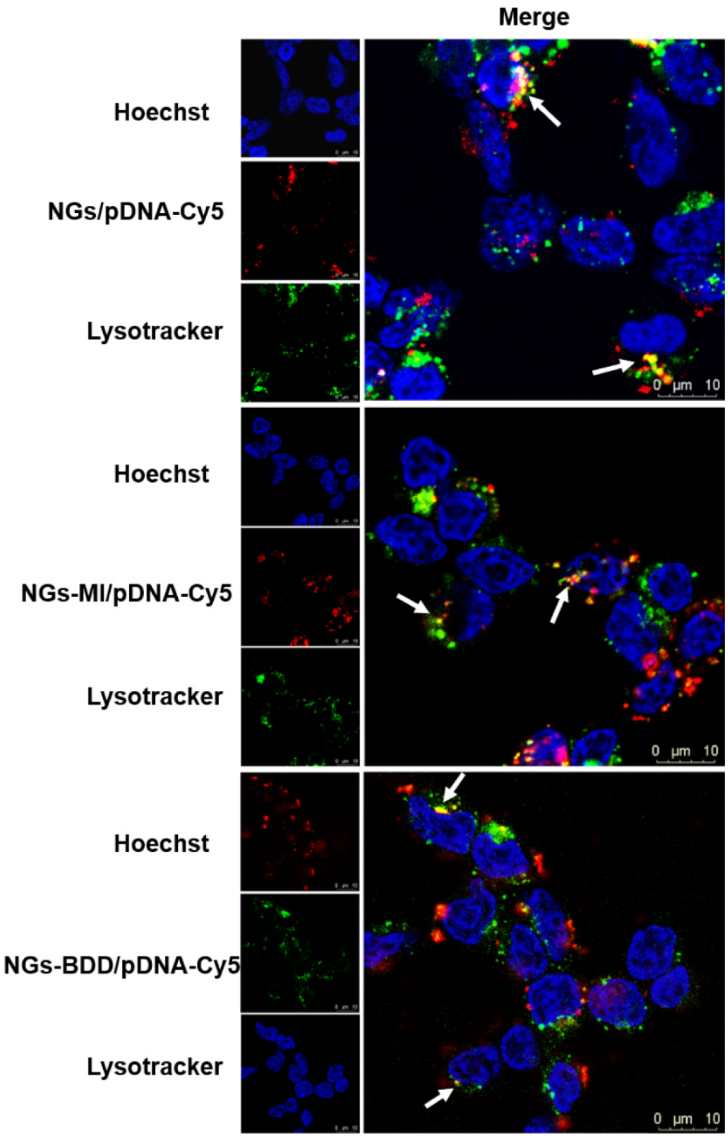

Figure S5. Intracellular localization of Cy5-labeled pDNA (red) in HEK293 cells incubated with nanogel/Cy5-pDNA complexes, observed by CLSM at 4 h post incubation. The cell nuclei were stained with Hoechst33324 (blue) and lysosomes were stained with LysoTracker Green DND-22 (green). White arrows indicate colocalization between Cy-5 DNA and Lysotracker fluorescence signals. Scale bars indicate 10 µm.

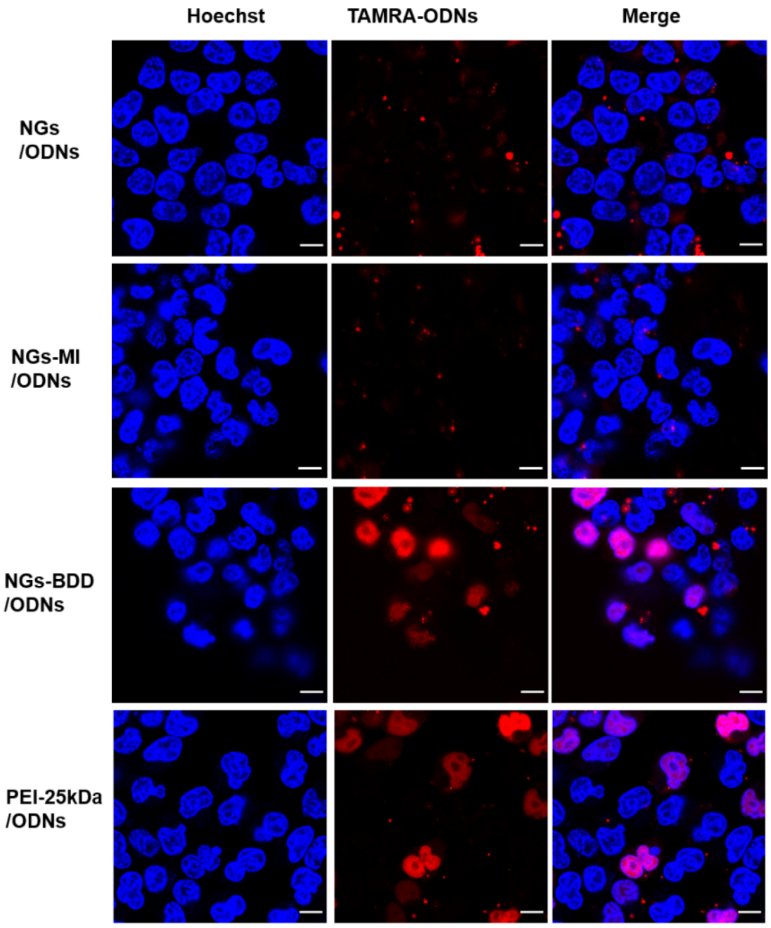

Figure S6. CLSM images of HEK293 cells after incubation with fluorescently labeled NGs/ODNs, NGs-MI/ODNs, NGs-BDD/ODNs and PEI-25kDa/ODNs for 4 h. TAMRA-labeled ODNs (red) were used. Cell nuclei were stained with Hoechst33324 (blue). Scale bars indicate 10 μm.
